# Complete mitogenome assemblies from a panel of 13 diverse potato taxa

**DOI:** 10.1080/23802359.2021.1886016

**Published:** 2021-03-15

**Authors:** Sai Reddy Achakkagari, Ilayda Bozan, Noelle L. Anglin, David Ellis, Helen H. Tai, Martina V. Strömvik

**Affiliations:** a Department of Plant Science, McGill University, Montreal, Canada; b International Potato Center, Lima, Peru; c Fredericton Research and Development Centre, Agriculture and Agri-Food Canada, Fredericton, Canada

**Keywords:** Potato, mitogenome, mtDNA assembly, *Solanum*

## Abstract

Mitochondrial DNA is maternally inherited and is shown to affect nuclear–cytoplasmic interactions in potato. Analyzing the mitogenome helps understand the evolutionary relationships and improve breeding programs in potato. We report complete mitogenome sequences from a panel of 13 potato accessions of various taxa. Each mitogenome has three independent circular molecules, except one of the *S. bukasovii* sample BUK2, which has a single circular molecule. Each mitogenome code for 37 non-redundant protein-coding genes, three rRNAs, 20 tRNAs, and 19 hypothetical open reading frames. Phylogenetic analysis reveals congruency between plastome and mitogenome phylogeny.

## Introduction

Plant mitochondrial genomes, mitogenomes, are larger and more complex compared to those of other eukaryotic organisms (Varré et al. 2019). The structure and organization of potato mitogenomes include circular and linear conformations with sub-genomic molecules generated by recombination events at repeat regions (Cho et al. 2017; Varré et al. 2019). In addition to functions in common with other organisms’ mitochondria, such as respiration, metabolism, and programmed cell death, plant mitochondria also have a function in male fertility (Kozik et al. 2019). Mitochondrial genomes show divergent evolution that affects nuclear–cytoplasmic interactions (Grun 1990). These interactions are important in breeding when intercrossing between related *Solanum* species, which are riddled with incompatibles. However, complete mitogenome sequences have only been reported for two potato species, *S. tuberosum* and *S. commersonii*, to date (Cho et al. 2017, 2018; Varré et al. 2019). Improving genetic resources helps to better understand the genetic diversity within potato. The current study examines complete mitochondrial genome sequences from 13 accessions of diverse tuber-bearing *Solanum* taxa (Achakkagari et al. 2020).

## Materials and methods

### DNA extraction and sequencing

Total genomic DNA was extracted from the leaves of 13 potato accessions (*Solanum stenotomum* subsp. *goniocalyx* (GON1) (CIP 2021a), *S. stenotomum* subsp. *goniocalyx* (GON2) (CIP 2021b), *S. phureja* (PHU) (CIP 2021c), *S. xajanhuiri* (AJH) (CIP 2021d), *S. stenotomum* subsp. *stenotomum* (STN) (CIP 2021e), *S. bukasovii* (BUK1) (CIP 2021f), *S. bukasovii* (BUK2) (CIP 2021g), *S. tuberosum* subsp. *andigena* (ADG1) (CIP 2021h), *S. tuberosum* subsp. *andigena* (ADG2) (CIP 2021i), *S. curtilobum* (CUR) (CIP 2021j), *S. juzepczukii* (JUZ) (CIP 2021k), *S. chaucha* (CHA) (CIP 2021l), and *S. tuberosum* subsp. *tuberosum* (TBR) (CIP 2021m)) grown at the International Potato Center (CIP) in Lima, Peru as described (Kyriakidou et al. 2020), and sequenced using Illumina PE technology, except BUK2, which was sequenced with 10X Genomics’ GemCode technology (https://www.10xgenomics.com/).

### Mitogenome assembly and annotation

The raw reads obtained from Illumina sequencing were initially processed using Trimmomatic v0.39 (Bolger et al. 2014). The parameters used for the Trimmomatic are ILLUMINACLIP: TruSeq3-PE. Fa: 2:30:10 LEADING:3 TRAILING:3 SLIDINGWINDOW: 4:15 MINLEN:60. The reads of BUK2 obtained from the 10X Genomics’ GemCode technology were trimmed using a python script ‘filter_10xReads.py’ (Davis 2021). Potato mitochondrial genome sequences available from the GenBank were downloaded and concatenated to create a reference set (GenBank accession no: *MF989953.1*, *MF989954.1*, *MF989955.1*, *MF989956.1*, *MF989957.1*, *MF989958.1*, *MF989959.1*, *MF989960.1*, *MF989961.1*, *MN1044801.1*, *MN104802.1*, *MN104803.1*, *MN114537.1*, *MN114537.2*, and *MN114537.3*) (Cho et al. 2017; Varré et al. 2019). The filtered reads of each mitogenome from the CIP panel of potato species were mapped against this reference set. The mapped reads were extracted and used in an initial assembly with GetOrganelle (Jin et al. 2019). The longest contigs in the assembly were selected based on *Blast* searches against the reference set and then used as seed contigs for further assembly (Johnson et al. 2008). The seed contigs were extended iteratively to get a complete mitogenome sequence using NOVOPlasty v4.2 (Dierckxsens et al. 2016). Filtered WGS reads were used for the extension to retain unique mitochondrial sequences. For BUK2, an initial assembly was carried out using NOVOPlasty v4.2 with filtered reads (Dierckxsens et al. 2016). Then, filtered reads of BUK2 were mapped to the reference set along with NOVOPlasty-assembled contigs. The fastq files suited for supernova were generated from the mapped reads using a python script ‘regen_10xReads.py’ (Davis 2021), and executed supernova with *–accept-extreme-coverage* option (Weisenfeld et al. 2017). The supernova-assembled scaffolds along with the NOVOPlasty-assembled contigs were run through tigmint, arcs, and LINKS to correct misassemblies and to further assemble into scaffolds (Warren et al. 2015; Yeo et al. 2017; Jackman et al. 2018). The assembled sequences were checked for structural errors using NucBreak (Khelik et al. 2020), and then annotated using GeSeq with the reference set of mitogenomes (Tillich et al. 2017). The annotations were manually examined and curated using BLAST searches (Johnson et al. 2008).

### Phylogeny

The coding sequences of common protein-coding genes (*atp4*, *atp8*, *atp9*, *ccmB*, *ccmC*, *ccmFc*, *ccmFn*, *cob*, *cox1*, *cox2*, *cox3*, *matR*, *mttB*, *nad3*, *nad4L*, *nad6*, *nad7*, *nad9*, *rpl10*, *rpl2*, *rpl5*, *rps12*, *rps13*, *rps19*, *rps3*, *rps4*, *sdh3*, *sdh4*) were extracted and concatenated from each mitogenome, and used to construct a phylogenetic tree (Varré et al. 2019). The reference mitogenomes used in this study were also added to the phylogeny. Sequences were then aligned using MAFFT v7.471 (Katoh et al. 2002) and a maximum parsimony phylogenetic tree was constructed using MEGA-X (Kumar et al. 2018) with 1000 bootstrap replicates.

## Results and discussion

### Mitogenome assembly

The mitogenome of each of the 13 CIP potato accessions was assembled and the results show that they range in size from 429,483 bp to 478,227 bp (GenBank accession MW122949–MW122985). Each mitogenome was assembled into three independent circular molecules, except BUK2, which was assembled into a single circular molecule (Table 1). Molecule 1 ranges from 49,155 bp to 49,302 bp between 12 mitogenomes. Similarly, molecule 2 and 3 range from 111,694 bp to 113,545 bp and 284,372 bp to 316,322 bp, respectively. BUK2 was excluded since it does not have a discernible molecule 1, 2, and 3. Plant mitochondria are very complex, and recombination events lead to the presence of alternative arrangements. Previous studies have also reported similar mitogenome conformations in potato species. Five circular DNA molecules were reported in *S. tuberosum* (Cho et al. 2017), two circular DNA molecules in *S. commersonii* (Cho et al. 2018), and recently two circular and one linear conformation were observed in two *S. tuberosum* cultivars (Varré et al. 2019). Numerous direct/inverted repeat sequences are present in each mitogenome. A few repeat sequences are larger than 1000 bp and are conserved between the mitogenomes. The largest repeat sequence ranges from 11,309 bp to 11,916 bp, and is present in all of the 13 mitogenomes (Table 2). The smallest repeat sequence is 1589 bp, and is present in all of the mitogenomes, except BUK2. The repeat R2 is present only in ADG1, ADG2, and CHA. Similarly, the R4 repeat is only present in GON1, GON2, PHU, STN, BUK1, and CUR, mitogenomes where the repeat structure is generally the same. The repeat structure in ADG2 and CHA, AJH and JUZ is also generally the same. A previous study reported that five repeats, which are larger than 1000 bp are present in two of the *S. tuberosum* cultivars (Varré et al. 2019). The repeats R1, R3, R4, and R5 mentioned below were found in these two cultivars as well. However, a 1208 bp repeat present in these two cultivars is missing in all the 13 CIP mitogenomes in the present study.

**Table 1.** Conformation and size of the observed molecules present in the mitogenomes of 13 potato cultivars.

Potato cultivar	Total mitogenome size (bp)	Molecule 1 (bp)	Molecule 2 (bp)	Molecule 3 (bp)	Sequence coverage (X) on mitogenome
GON1	446,946	49,155	113,419	284,372	2299
GON2	446,946	49,155	113,419	284,372	2518
AJH	450,287	49,272	113,417	287,598	1876
PHU	446,946	49,155	113,419	284,372	1859
STN	446,946	49,155	113,419	284,372	1568
BUK1	446,947	49,155	113,419	284,373	2037
ADG1	478,227	49,273	112,632	316,322	1214
ADG2	474,612	49,302	113,450	311,860	1920
JUZ	455,624	49,236	113,545	292,843	1418
CHA	474,615	49,302	113,453	311,860	1485
CUR	446,162	49,155	112,635	284,372	1533
TBR	473,790	49,254	111,694	312,842	2064
BUK2	429,483	–	–	–	1029

Each mitogenome has three independent circular molecules with varying size, except BUK2. BUK2 mitogenome has one master circle with 429,483 bp in size.

**Table 2.** Repeats and their size in each mitogenome.

Potato cultivar	R1 (repeat)	R2 (repeat)	R3 (repeat)	R4 (repeat)	R5 (repeat)
GON1	11,909	–	–	4513	1589
GON2	11,909	–	–	4513	1589
AJH	11,909	–	7502	–	1589
PHU	11,909	–	–	4513	1589
STN	11,909	–	–	4513	1589
BUK1	11,909	–	–	4513	1589
ADG1	11,909	10,203	–	–	1589
ADG2	11,916	10,203	7494	–	1589
JUZ	11,909	–	7502	–	1589
CHA	11,916	10,203	7494	–	1589
CUR	11,909	–	–	4513	1589
TBR	11,909	–	7502 7232 7008	–	1589
BUK2	11,309	–	1234	–	–

Presence of repeats that are larger than 1000 bp are reported here. Each mitogenome has two copies of the R1 repeat sequence ranging from 11,309 bp to 11,916 bp. Similarly, each mitogenome has two copies of the R5 repeat sequence of 1589 bp, except BUK2. Two copies of R2, R3, and R4 repeat sequences are present only in the mentioned genomes. The TBR mitogenome has three copies of R3 repeat sequence with reduction in size in its second and third copy. The R3 repeat sequence in BUK2 is 1234 bp only.

### Mitogenome annotation

The 13 mitogenomes were annotated to determine their gene content and organization. Each encode 37 non-redundant protein-coding genes, three rRNAs, and 20 tRNAs. In addition, each mitogenome has 19 non-redundant hypothetical open reading frames. The majority of mitogenomes had internal stop codons in *orf111* (GON1, GON2, PHU, STN, BUK1, ADG1, ADG2, JUZ, and CUR). Similarly, *orf140* in BUK2 contained internal stop codons, whereas *orf123* in the TBR mitogenome was truncated at the 5′ end. A pseudogene *rps14* and a truncated copy of the *cob* gene are present in all the genomes, except BUK2. The arrangement of Ψ*rps14-*Ψ*cob* was previously observed in *S. tuberosum* accessions (Varré et al. 2019). Due to the presence of repeats that contained duplicated genes, the AJH, ADG1, ADG2, JUZ, CHA, and TBR mitogenomes have a higher number of total genes compared to the rest; however, the unique set of genes remains the same among all of them. The genes *cox2*, *rpl16*, *rps19*, *rps3*, and *orf102* are duplicated in AJH, JUZ, CHA, ADG2, and TBR mitogenomes due to the presence of the R3 repeat sequence. This duplication was also reported in two *S. tuberosum* cultivars (Varré et al. 2019). Similarly, *orf131* is duplicated in ADG1, ADG2, and CHA due to the presence of the R2 repeat sequence. A ribosomal protein *rps1* is duplicated only in BUK2.

### Phylogenetic analysis

**Figure 1.**
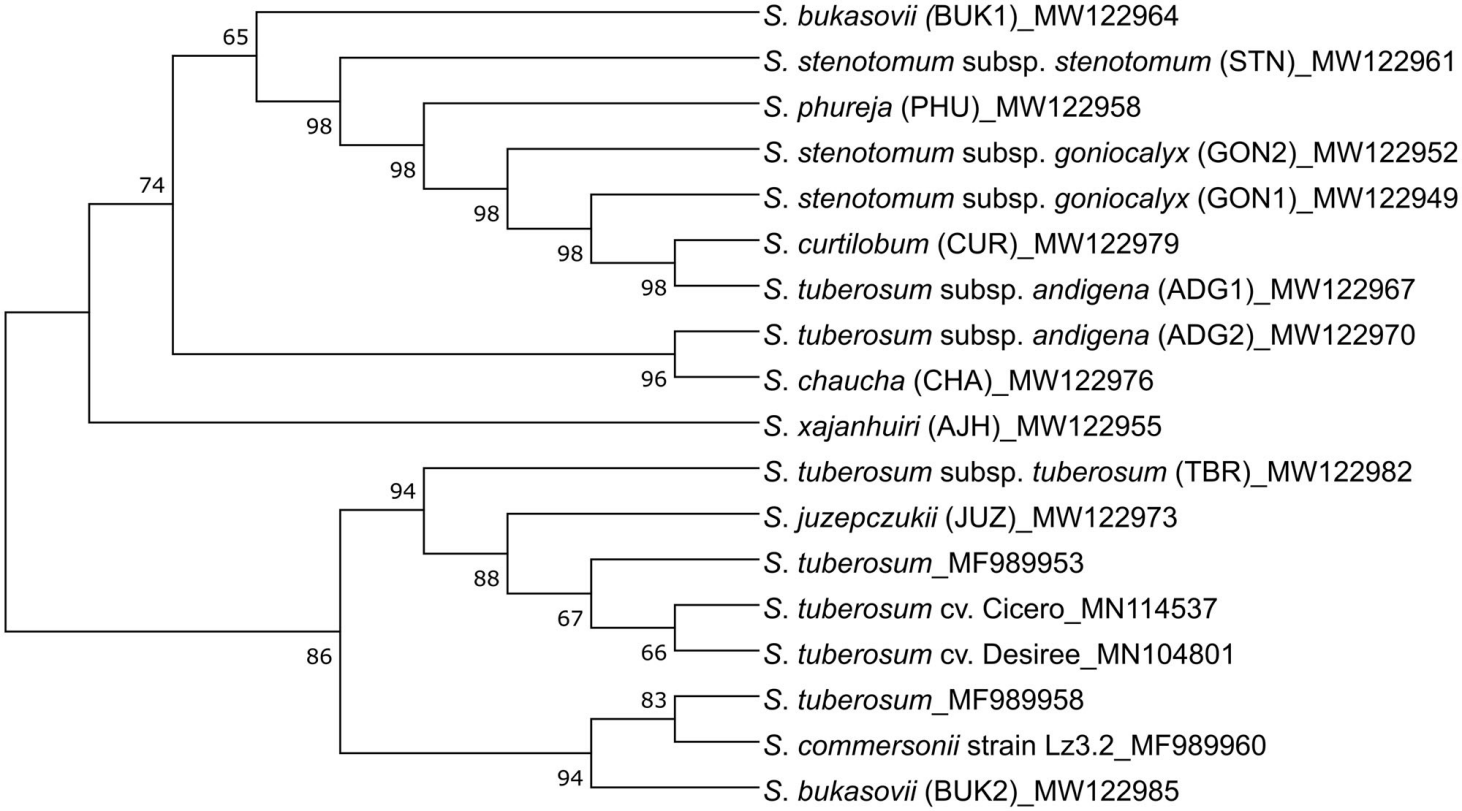
The Phylogenetic tree of 18 mitogenomes from a range of *Solanum* species (cultivated and wild). The GenBank accession of each mitogenome is provided in the figure.

Mitochondrial DNA is maternally inherited and can be used to accurately identify evolutionary relationships. Generating nuclear phylogeny along with organelle phylogeny can be an effective help in understanding the history of hybridization (Achakkagari et al. 2020). From the phylogenetic reconstruction, it was observed that GON1, GON2, PHU, STN, BUK1, ADG1, and CUR accessions are grouped together with no significant genetic variation (Figure 1). A previous study reported a similar classification for the *S. stenotomum* subsp. *goniocalyx*, *S. phureja*, and *S. stenotomum* subsp. *stenotomum* species based on six mitogenome markers (Bonen et al. 2007). Similarly, ADG2 and CHA mitogenomes are grouped together. The nuclear and plastome phylogeny of these 13 accessions was previously determined (Achakkagari et al. 2020; Kyriakidou et al. 2020), and a similar type of grouping was observed in these potato accessions. Also, a similar grouping of AJH, JUZ, and TBR accessions was observed in the plastome phylogeny. It is interesting to see all the *S. tuberosum* species in one clade, except for ADG1 and ADG2. The BUK2, which was previously observed to have close relations with wild species, is grouped with *S. commersonii* here. The two accessions of *S. bukasovii* (BUK1 and BUK2) are phylogenetically distant from each other. Similar results were observed in a previous study, and it is likely due to the collection of this accession as a natural population (Achakkagari et al. 2020). The reference species are confined to a single clade in this phylogeny; however, the accessions from our panel are widespread across the phylogeny, representing a wider variety of *Solanum* taxa.

## Conclusions

The mitogenome of 13 potato accessions from a selected panel was assembled and annotated. Three independent circular conformations were observed in all the accessions, except BUK2. The repeat structure in these mitogenomes is interesting and varies from accession to accession. The core genes are similar in all the accessions; however, the AJH, ADG1, ADG2, JUZ, CHA, and TBR mitogenomes have duplicated genes resulting from the repeat sequences. Finally, the phylogenetic relationships between these species were determined. The clustering of these species is mostly in agreement with the previous studies. This is the first study that reports complete mitogenome sequences from a panel that represents seven species, nine taxa, and two wild relatives based on Hawkes taxonomy (Hawkes 1990). The results of this study will greatly improve the genetic resources of potato mitogenome. It will also be useful in future comparative studies to better understand the evolutionary relationships in potato species.

## Data Availability

The genome sequence data that support the findings of this study are openly available in GenBank of NCBI at https://www.ncbi.nlm.nih.gov/nuccore/ under the accession numbers MW122949–MW122985. The associated BioProject number is PRJNA556263, SRA accession numbers are SRR10248510–SRR10248515, SRR10244436–SRR10244441, and BioSample numbers are SAMN12684886–SAMN12684896, SAMN12345900.
